# Patient Satisfaction With Digital Versus Conventional Impression Techniques in Clear Aligner Therapy: A Questionnaire-Based Comparative Study

**DOI:** 10.7759/cureus.107027

**Published:** 2026-04-14

**Authors:** Althaf Muhammed, Benoy Ambooken, Reshma Harikrishnan, Layana Theadore, Mahesh Kumar B, Rahul Tiwari

**Affiliations:** 1 Department of Dentistry, TOVALIGN Aligners, Kochi, IND; 2 Department of Orthodontics, TOVALIGN Aligners, Kochi, IND; 3 Department of Prosthodontics, TOVALIGN Aligners, Kochi, IND; 4 Department of Orthodontics, Dr. Udhayaraja's Dental and Orthodontic Centre, Chennai, IND; 5 Department of Research Cell, Dr. D. Y. Patil Dental College and Hospital, Dr. D. Y. Patil Vidyapeeth (Deemed to be University), Pune, IND

**Keywords:** impression technique, intraoral scanners, orthodontic appliances, orthodontics, patient satisfaction

## Abstract

Introduction

Clear aligner therapy has gained popularity because of its esthetic appeal and patient-friendly nature. The impression-making process plays a crucial role in shaping a patient’s experience and treatment acceptance. With the increasing use of digital technologies, it is important to evaluate whether digital impressions improve patient satisfaction compared with conventional techniques. The aim of this study was to compare patient satisfaction between digital and conventional impression techniques and to evaluate patient-reported comfort and fit of aligners fabricated using these methods.

Materials and methods

This prospective questionnaire-based study included 50 patients aged 13-30 years who required mild orthodontic correction. Each participant underwent both digital impressions using an intraoral scanner and conventional impressions using polyvinyl siloxane on separate occasions. Clear aligners were fabricated using both techniques, and patient satisfaction was assessed using a validated self-designed questionnaire. The evaluated parameters included comfort, breathing difficulty, gag reflex, anxiety, perceived duration, aligner fit, distortion, and ease of speech. Data were analyzed using non-parametric tests, with statistical significance set at p < 0.05.

Results

Digital impression techniques demonstrated significantly higher patient satisfaction across all evaluated parameters, including comfort, pleasantness, breathing ease, absence of gag reflex, and overall experience (p = 0.001 for most comparisons). Patients also showed greater willingness for repetition with digital methods (p = 0.005). Digitally fabricated aligners were associated with significantly better tray comfort, fit, and reduced distortion (p = 0.001). However, no statistically significant difference was observed between digital and conventional techniques in terms of ease of speech (p = 0.062). Overall satisfaction scores were significantly higher for the digital workflow (p = 0.001).

Conclusion

Digital impression techniques significantly enhanced patient satisfaction and acceptance of clear aligner therapy. Improved comfort during impression-making and a better perceived aligner fit may contribute to increased compliance. Incorporating digital workflows into orthodontic practice can optimize patient-centered outcomes and improve the overall treatment experience.

## Introduction

The increasing demand for esthetic and patient-friendly orthodontic treatment options has led to the rapid evolution of clear aligner therapy. Unlike conventional fixed appliances, clear aligners offer advantages such as improved esthetics, removability, and enhanced oral hygiene maintenance, making them highly acceptable to patients [1]. However, the success of aligner therapy is solely dependent not only on biomechanical efficiency but also on patient experience during various stages of treatment, particularly the impression-making process [2,3]. Because impressions serve as the foundation for aligner fabrication, the technique used can significantly influence patient perception, comfort, and overall satisfaction.

Traditionally, conventional impressions using elastomeric materials, such as polyvinyl siloxane (PVS), have been widely employed because of their ability to capture fine anatomical details. Despite their accuracy, these techniques are often associated with patient discomfort, including gag reflex, breathing difficulty, unpleasant taste, and longer chairside time. These factors can negatively impact patient cooperation and satisfaction, especially in younger or anxious individuals [4].

With advancements in digital dentistry, intraoral scanning has emerged as a modern alternative for recording the dentogingival structures. Digital impressions offer several potential advantages, including reduced chairside time, elimination of impression materials, improved patient comfort, and real-time visualization [5]. These features may significantly enhance patient acceptance and clinical efficacy. Joshi et al. [6] demonstrated that both conventional and digital impression techniques can achieve clinically acceptable precision and marginal fit, although differences exist depending on workflow and technique sensitivity.

Their findings highlight the clinical relevance of comparing these techniques, supporting the need to evaluate patient-centered outcomes, such as comfort and satisfaction, as addressed in the present study. This study aimed to compare patient satisfaction between conventional and digital impression techniques in clear aligner therapy. The objectives of this study were to evaluate patient comfort, perception of procedure duration, breathing difficulty, gag reflex, and overall acceptance using a structured questionnaire.

## Materials and methods

This prospective, questionnaire-based clinical study was conducted over a period of six months from January 2024 to June 2024 among 50 patients aged 13-30 years who required mild orthodontic correction in the maxillary arch at the RKDF Dental College and Research Centre, Bhopal, Madhya Pradesh, India. Ethical approval for this study was obtained from the Institutional Ethics Committee of the College (Approval No.: RKDF/DC/PG/2024/SS-09). This study was conducted in accordance with the principles of the Declaration of Helsinki. Written informed consent was obtained from all the participants prior to their inclusion in the study. Only patients with complete permanent dentition and healthy periodontal status were included, whereas those with systemic diseases, craniofacial anomalies, or previous orthodontic treatment were excluded. Each participant underwent both digital and conventional impression procedures, allowing for an intraindividual comparison of patient satisfaction.

The sample size for this study was determined using G*Power software, version 3.1 (Heinrich Heine University, Düsseldorf, Germany), based on an a priori power analysis for comparison between the two groups. A moderate effect size of 0.35 was assumed based on findings from a previous study [7]. The level of significance (α) was set at 0.05, and statistical power (1−β) was fixed at 80%. The analysis indicated that a minimum of 46 samples were required to detect a statistically significant difference. The sample size was increased to 50 participants to account for potential dropouts and incomplete responses.

Digital impressions were obtained using an intraoral scanner (Shining 3D Technology Co., Ltd., Hangzhou, China), which captured real-time three-dimensional images of the dentogingival structures. The acquired data were saved as standard tessellation language (STL) files following manufacturer-recommended protocols. Conventional impressions were made using a PVS impression material (3M ESPE, St. Paul, Minnesota, USA) with appropriately selected perforated trays under standardized clinical conditions. To minimize procedural bias, both impression techniques were performed on separate days. Clear aligner trays were fabricated from both digital and conventional models using clear aligners (TOVALIGN Aligners, Kochi, Kerala, India), ensuring uniformity in the materials and fabrication procedures. Each aligner was inserted intraorally, and the patients were asked to wear them briefly to assess their comfort before providing feedback.

Both digital and conventional impression procedures were performed on separate days, with a washout period of two to three days between them to minimize recall bias. Following fabrication, the aligners (one digital, one conventional) were inserted intraorally in a randomized sequence during two separate visits; thus, the aligners were used consecutively rather than simultaneously. At each visit, the patient was instructed to wear the aligner for approximately 15-20 minutes under clinical supervision to assess immediate comfort, fit, retention, speech, and overall perception. After removal, the patient completed the corresponding section of the questionnaire. The second aligner was evaluated in a similar manner during the subsequent visit, ensuring independent assessment of each technique.

Patient-reported outcomes were assessed using a self-designed structured questionnaire comprising two sections (Appendices). The first section evaluated the experience of impression-making, including comfort, gag reflex, breathing difficulty, anxiety, and perceived duration of the procedure. The second section assessed patient perception of the aligners, including overall comfort, ease of speech, retention, and perceived fit/adaptation of the tray to the teeth. Responses were recorded on a 5-point Likert scale (1 = strongly agree to 5 = strongly disagree), with higher scores indicating greater discomfort or dissatisfaction. Domain-wise and total scores were calculated by summing individual item scores, with lower scores representing a more favorable patient experience.

The questionnaire was validated by a panel of orthodontic experts to ensure relevance and clarity, and face validity was established through preliminary testing. A pilot study was conducted on 10 patients to refine the questionnaire and assess its feasibility; these participants were excluded from the final analysis. Reliability testing demonstrated acceptable internal consistency using Cronbach’s alpha (α ≥ 0.7), and test-retest reliability confirmed the reproducibility of the responses.

All data were analyzed using the Statistical Package for the Social Sciences (SPSS) software (version 26.0, IBM Corp., Armonk, New York, USA). The normality of the data distribution was assessed using the Shapiro-Wilk test. As the data obtained from the questionnaire were ordinal, non-parametric tests were applied. Descriptive statistics were expressed as mean, standard deviation, median, and range for continuous variables and as frequencies and percentages for categorical variables. Intergroup comparisons between the digital and conventional techniques were performed using the Mann-Whitney U test. Statistical significance was set at p < 0.05.

## Results

The study included 50 participants, with a mean age of 24.56 ± 6.70 years. The study population demonstrated a slight female predominance compared to male predominance, representing a typical orthodontic patient demographic (Table 1).

**Table 1:** Demographic details of the study population (n = 50). Values are expressed as mean ± SD for continuous variables and as number (percentage) for categorical variables. SD, standard deviation

Parameter	Category	Unit	Value
Age (years)	Male	Mean ± SD	25.34 ± 6.12
	Female	Mean ± SD	23.42 ± 5.81
	Overall	Mean ± SD	24.56 ± 6.70
Sex	Male	n (%)	22 (44)
	Female	n (%)	28 (56)

Patient perceptions of impression techniques showed a statistically significant difference between the digital and conventional methods across all evaluated parameters (Table 2). Digital impressions were consistently rated favorably in terms of overall comfort, reduced unpleasant taste, improved breathing ease, and minimal gag reflex. Patients also perceived digital procedures as less time-consuming and more convenient. The overall perception scores indicated a significantly better patient experience with digital impressions than with conventional techniques (p < 0.05).

**Table 2:** Comparison of patient perception for impression techniques. Values are expressed as median (interquartile range: Q1–Q3). The Mann–Whitney U test was used for intergroup comparison, and responses were recorded using a 5-point Likert scale (1 = strongly agree to 5 = strongly disagree). *A p-value of <0.05 indicates statistical significance.

Parameter	Group	Median (lower quartile–upper quartile)	U	p-Value
Comfort	Digital	2 (1–3)	13	0.001*
	Conventional	3 (3–5)		
Pleasant	Digital	1 (1–2)	2.5	0.001*
	Conventional	4 (2–5)		
Repetition	Digital	4 (2–5)	178	0.005*
	Conventional	3 (2–4)		
Less appointments	Digital	1 (1–3)	54	0.001*
	Conventional	3 (2–4)		
Easy breathing	Digital	2 (1–2)	16	0.001*
	Conventional	3 (3–4)		
No gag reflex	Digital	2 (1–2)	5.5	0.001*
	Conventional	4 (3–5)		
Total score	Digital	10 (8–14)	25.63	0.001*
	Conventional	21 (13–16)		

With respect to patient-reported outcomes for aligner use, digitally fabricated aligners were associated with significantly higher satisfaction levels for most of the parameters (Table 3). The patients reported improved comfort, better adaptation of the tray to the teeth, and reduced perception of distortion with digitally derived aligners. However, no statistically significant difference was observed between the two groups in terms of ease of speech, indicating a comparable performance in this aspect. Overall, the total satisfaction scores favored digital aligners, reflecting enhanced patient acceptance (p < 0.05).

**Table 3:** Comparison of patient perception for aligner performance. Values are expressed as median (interquartile range: Q1–Q3). The Mann–Whitney U test was used for intergroup comparison, and responses were recorded using a 5-point Likert scale (1 = strongly agree to 5 = strongly disagree). *A p-value of <0.05 indicates statistical significance

Parameter	Group	Median (lower quartile–upper quartile)	U	p-Value
Tray comfort	Digital	2 (1–3)	162.5	0.001*
	Conventional	3 (2–3)		
Tray fit	Digital	1 (1–2)	107.5	0.001*
	Conventional	2 (1–3)		
Tray distortion	Digital	1 (1–2)	175	0.001*
	Conventional	2 (1–2)		
Easy speaking	Digital	3 (2–3)	231	0.062
	Conventional	3 (2–4)		
Total	Digital	7 (5–9)	7.42	0.001*
	Conventional	9 (7–11)		

## Discussion

The present study evaluated patient satisfaction with digital and conventional impression techniques in the context of clear aligner therapy, with additional emphasis on patient-reported perceptions of aligner comfort and fit. The findings demonstrated a clear preference for digital impressions, with significantly improved scores across multiple domains, including comfort, ease of breathing, reduced gag reflex, and overall experience. These results reinforce the growing evidence that patient-centered outcomes play a crucial role in the acceptance and success of orthodontic treatment.

Digital impressions were consistently rated as superior to conventional methods in terms of patient comfort and procedural acceptability. This can be attributed to the elimination of bulky impression trays and impression materials, which are often associated with discomfort, unpleasant tastes, and gag reflexes. Similar findings were reported by Burzynski et al. [8], who observed that patients preferred intraoral scanning owing to reduced discomfort and shorter procedure duration. Likewise, Yuzbasioglu et al. [5] demonstrated that digital impression techniques significantly improved patient comfort and reduced anxiety compared with conventional methods. Burhardt et al. [9] reported that digital impression techniques were associated with significantly greater patient comfort, reduced perceived treatment time, and higher preference than conventional methods, particularly among younger patients. The present study aligns with these observations, further confirming that digital workflows enhance overall patient experience during the initial stages of aligner therapy.

Breathing difficulty and gag reflex are well-documented limitations of conventional impression techniques, particularly in patients with heightened gag reflex sensitivity. In the present study, patients reported significantly better breathing comfort and minimal gag reflex with digital impressions. These findings are consistent with previous studies, which highlight that intraoral scanners minimize intraoral obstruction and allow patients to breathe normally during the procedure [10]. This improvement in patient tolerance is clinically relevant as it may reduce procedure-related anxiety and improve cooperation, especially in younger or apprehensive patients.

Interestingly, although digital impressions were preferred overall, willingness to repeat procedures showed a relatively different trend, which may reflect patient familiarity with conventional methods or apprehension toward newer technologies. This highlights the importance of patient education and clinician communication when introducing digital workflow into routine practice.

In addition to impression techniques, the present study evaluated patient-reported outcomes related to aligner comfort and fit. Digitally fabricated aligners are associated with significantly better patient satisfaction in terms of comfort, perceived fit, and reduced distortion [11]. These findings suggest that improved data acquisition and streamlined digital workflows may contribute to enhanced adaptation of the aligner to dentition. Previous studies have reported that digital impressions can improve the precision of appliance fabrication by eliminating intermediate steps, such as impression material handling and cast pouring, thereby reducing cumulative errors [5,8,10].

The perception of improved aligner fit in the digital group, as reported by patients, is particularly noteworthy. Although objective measurements of fit were not the primary focus of this analysis, patient perception remained a critical determinant of compliance with aligner therapy. A well-fitting aligner is more likely to be comfortable, less intrusive during speech, and acceptable for prolonged wear. Patzelt et al. [12] demonstrated that digitally fabricated appliances exhibited improved efficiency and clinically acceptable fit compared to their conventionally fabricated counterparts, supporting the advantages of digital workflows in dental appliance fabrication. Cheng et al. [13] demonstrated that digital workflows improved treatment efficiency while providing a comparable or superior fit of interim crowns compared to conventional methods, irrespective of clinician experience.

However, no significant difference was observed between the groups in terms of the ease of speech. This suggests that factors such as aligner material thickness, design, and coverage may have a greater influence on speech than impression technique. Similar observations have been reported in the literature, indicating that speech adaptation is multifactorial and not solely dependent on fabrication technique [14]. Overall, the findings of this study highlight that digital impression techniques not only improve patient experience during impression-making but also positively influence patient perception of aligner comfort and fit. Given that patient compliance is a key determinant of treatment success in clear aligner therapy, these advantages may translate to improved clinical outcomes.

Clinical implications

The results of this study suggest that the adoption of digital impression techniques can significantly enhance patient comfort and acceptance of orthodontic treatment. Improved patient experience during impression making may lead to better cooperation, reduced chairside time, and increased efficiency in clinical practice. Furthermore, the superior patient-reported comfort and fit of digitally fabricated aligners may contribute to improved compliance, which is critical for the success of clear aligner therapy. Clinicians should consider integrating digital workflows into routine practice to optimize both clinical efficiency and patient satisfaction.

Limitations

This study had certain limitations. The sample size was relatively small and limited to patients requiring mild orthodontic correction, which may have affected the generalizability of the findings. The evaluation of the aligner fit was based on subjective patient perception rather than objective quantitative measurements. Additionally, short-term assessments may not fully reflect long-term patient experiences and compliance. Future studies with larger sample sizes, inclusion of an objective fit analysis, and long-term follow-up are recommended to validate these findings.

## Conclusions

Within the limitations of this study, digital impression techniques demonstrated significantly higher patient satisfaction than conventional methods, particularly in terms of comfort, reduced gag reflex, improved breathing ease, and overall procedural acceptability. Additionally, aligners fabricated from digital impressions were perceived by patients to have a better fit, comfort, and reduced distortion, contributing to enhanced treatment acceptance. No significant difference was observed in speech, indicating that factors other than impression technique may influence phonetic adaptation. These findings highlight the importance of incorporating digital workflows in orthodontic practice to improve patient-centered outcomes and compliance, ultimately supporting more efficient and acceptable clear aligner therapy.

## Figures and Tables

**Figure 1:**
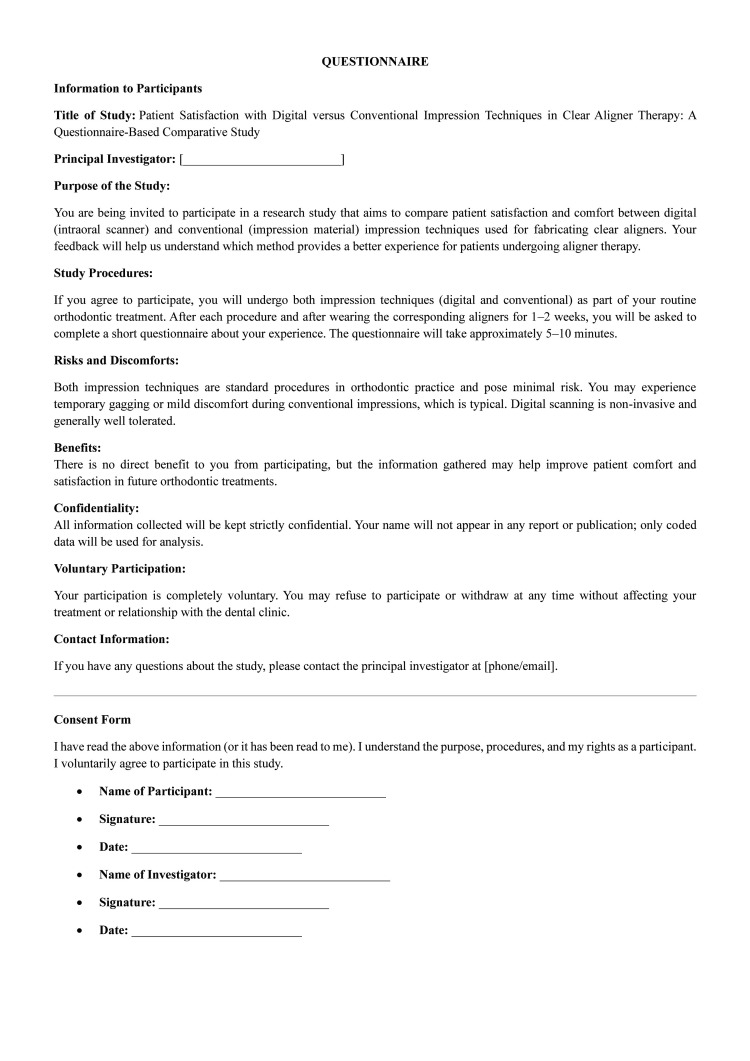
Questionnaire page 1 as patient information sheet. Credit: Althaf Muhammed, Benoy Ambooken, Reshma Harikrishnan.

**Figure 2:**
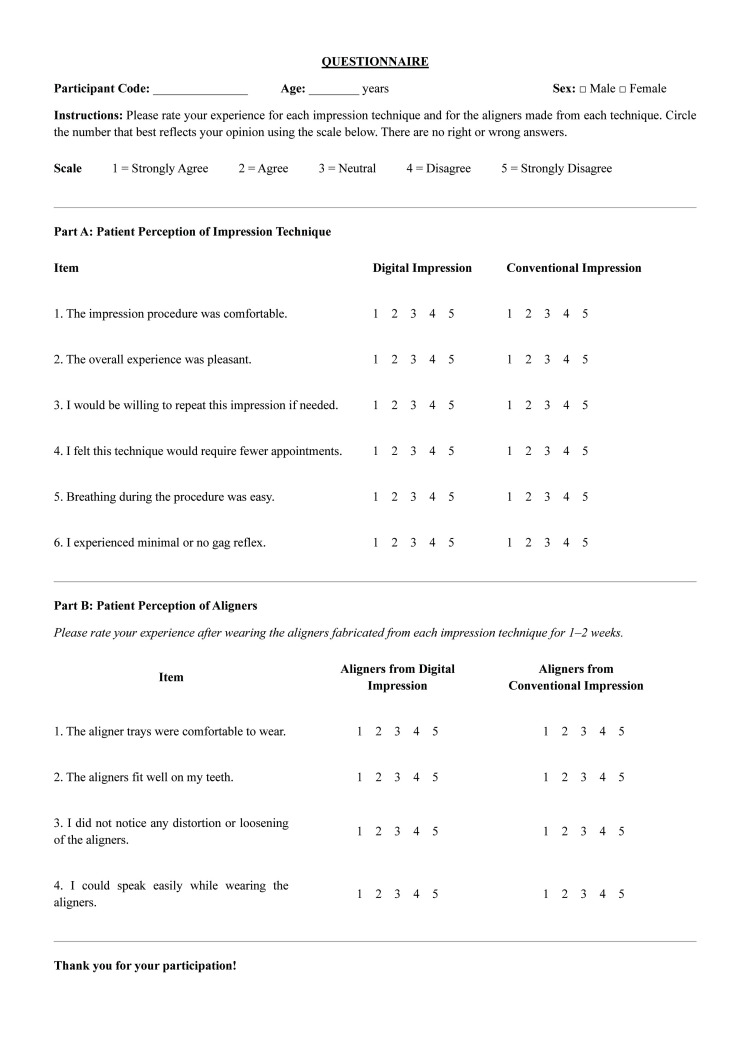
Questionnaire page 2 as patient response sheet. Credit: Althaf Muhammed, Benoy Ambooken, Reshma Harikrishnan.

## Appendices

Appendix A 

Appendix B 
